# Differential lipid signaling from CD4^+^ and CD8^+^ T cells contributes to type 1 diabetes development

**DOI:** 10.3389/fimmu.2024.1444639

**Published:** 2024-09-18

**Authors:** Tayleur D. White, Abdulaziz Almutairi, Ying Gai-Tusing, Daniel J. Stephenson, Benjamin D. Stephenson, Charles E. Chalfant, Xiaoyong Lei, Brian Lu, Bruce D. Hammock, Teresa P. DiLorenzo, Sasanka Ramanadham

**Affiliations:** ^1^ Department of Cell, Developmental, and Integrative Biology, Heersink School of Medicine, University of Alabama at Birmingham, Birmingham, AL, United States; ^2^ Comprehensive Diabetes Center, Heersink School of Medicine, University of Alabama at Birmingham, Birmingham, AL, United States; ^3^ Department of Basic Science, College of Science and Health Professions, King Saud bin Abdulaziz University for Health Sciences, King Abdullah International Medical Research Center, Riyadh, Saudi Arabia; ^4^ Cancer Biology Program, University of Virginia National Cancer Institute (UVA NCI) Comprehensive Cancer Center, University of Virginia-School of Medicine, Charlottesville, VA, United States; ^5^ Research Service, Richmond Veterans Administration Medical Center, Richmond, VA, United States; ^6^ Department of Medicine, University of Virginia-School of Medicine, Charlottesville, VA, United States; ^7^ Department of Cell Biology, University of Virginia-School of Medicine, Charlottesville, VA, United States; ^8^ Division of Endocrinology, Diabetes, and Metabolism, Department of Medicine, Heersink School of Medicine, University of Alabama at Birmingham, Birmingham, AL, United States; ^9^ Entomology and Nematology and Comprehensive Cancer Center, University of California, Davis, Davis, CA, United States; ^10^ Department of Microbiology and Immunology, Albert Einstein College of Medicine, New York, NY, United States

**Keywords:** T-lymphocytes, lipid signaling, type 1 diabetes, adoptive transfer, islet microscopy, flow cytometry, lipidomics

## Abstract

**Introduction:**

We reported that Ca^2+^-independent phospholipase A_2_β (iPLA_2_β)–derived lipids (iDLs) contribute to type 1 diabetes (T1D) onset. As CD4^+^ and CD8^+^ T cells are critical in promoting β-cell death, we tested the hypothesis that iDL signaling from these cells participates in T1D development.

**Methods:**

CD4^+^ and CD8^+^ T cells from wild-type non-obese diabetic (*NOD*) and *NOD*.*iPLA_2_β^+/-^* (NOD*.HET*) mice were administered in different combinations to immunodeficient NOD.*scid*.

**Results:**

In mice receiving only *NOD* T cells, T1D onset was rapid (5 weeks), incidence 100% by 20 weeks, and islets absent. In contrast, onset was delayed 1 week and incidence reduced 40%–50% in mice receiving combinations that included NOD*.HET* T cells. Consistently, islets from these non-diabetic mice were devoid of infiltrate and contained insulin-positive β-cells. Reduced iPLA_2_β led to decreased production of proinflammatory lipids from CD4^+^ T cells including prostaglandins and dihydroxyeicosatrienoic acids (DHETs), products of soluble epoxide hydrolase (sEH), and inhibition of their signaling decreased (by 82%) IFNγ^+^CD4^+^ cells abundance. However, only DHETs production was reduced from CD8^+^ T cells and was accompanied by decreases in *sEH* and *granzyme B*.

**Discussion:**

These findings suggest that differential select iDL signaling in CD4^+^ and CD8^+^ T cells contributes to T1D development, and that therapeutics targeting such signaling might be considered to counter T1D.

## Highlights

- iPLA_2_β-derived lipid (iDL) signaling participates in T1D development.
- T1D is a T-cell–mediated disease; however, the contribution of T-cell–derived lipid signaling to T1D development has not been studied.
- Herein, we sought to determine if iDL signaling in CD4^+^ and/or CD8^+^ T cells contributes to T1D development.
- We find that reducing iDL signaling in CD4^+^ and/or CD8^+^ T cells ameliorates disease progression and reduces T1D incidence.
- We posit that targeting select iDL signaling in T cells is a novel therapeutic avenue to counteract T1D onset.

## 1 Introduction

Type 1 diabetes (T1D) is a consequence of autoimmune destruction of pancreatic islet β-cells, involving activation of cellular immunity and inflammation initiated by early stage immune cell infiltration of islets (1, 2). It is well established that, in human subjects with T1D and non-obese spontaneous diabetes-prone (*NOD*) mice, both CD4^+^ and CD8^+^ T cells are major components of the islet infiltrate and critical contributors to T1D development (1, 3). First, autoreactive T cells are activated by β-cell antigens presented by antigen-presenting cells (APCs). The activated CD4^+^ T cells infiltrate the pancreas and are thought to contribute to β-cell destruction via activation of macrophages. The CD4^+^ T-cell–mediated β-cell destruction can be caused by the production of proinflammatory cytokines such as IFNγ and, indirectly, by activating local innate cells such as macrophages and dendritic cells to enhance infiltration. Moreover, activated T-helper cells are required to activate CD8^+^ T cells. Second, CD4^+^ T cells activate CD8^+^ T cells, which directly kill β-cells by interacting with MHC class I molecules and through perforin and granzyme secretion. It has been suggested that the MHC class I/CD8^+^ T-cell interaction is required for T1D in the early stages of development (4) and antigen presentation to CD4^+^ T cells within pancreatic islets is essential for β-cell destruction (3).

An integral component of T1D progression is an overall increase in the inflammatory status of T cells. It is well recognized that some lipid metabolites of arachidonic acid are profoundly inflammatory (5–10). However, their contribution to T1D development is understudied. Arachidonic acid residing at the *sn*-2 position of membrane glycerophospholipids is hydrolyzed by phospholipases A_2_ (PLA_2_s) (11) and subsequently metabolized by lipoxygenase (LOX), cyclooxygenase (COX), and cytochrome P450 (CYP450) enzymes to generate bioactive lipids, designated eicosanoids. Among the PLA_2_s is a cytosolic Ca^2+^-independent PLA_2_beta (iPLA_2_β) that is ubiquitously expressed, including in T cells (12). Upon stimulation, the iPLA_2_β translocates to subcellular organelles (13–15), where it manifests activity. We reported that the iPLA_2_β is induced during cytokine-mediated β-cell death and that inhibition or genetic reduction of iPLA_2_β mitigates β-cell apoptosis (14, 16–19), raising the possibility that iPLA_2_β-derived lipids (iDLs) contribute to β-cell death leading to T1D. Indeed, we demonstrated that global inhibition or reduction of iDL signaling in the *NOD* mouse significantly reduces insulitis, β-cell loss, and T1D incidence (12, 20). This strongly implied a role for iDLs in promoting T1D. In fact, temporal lipidomics analyses revealed increases in select iDLs prior to T1D onset (20), suggesting that they trigger inflammatory responses that induce β-cell death. The findings of a similar lipid signature in the plasmas of normoglycemic children at high risk for developing T1D support this possibility (20).

Proinflammatory iDLs have been reported to induce NFκB (21), nitric oxide (8), and cytokine genes and CCL2, also known as monocyte chemoattractant protein-1 (MCP-1) (22, 23). Furthermore, iDLs have been shown to participate in oxidative stress pathways (5), amplify ER stress (6, 24), and reduce inflammation-resolving processes (7, 10). They also impact T cells by promoting CD4^+^ Th1/Th17 differentiation (25) and modulating local activation of T cells (26).

To date, the potential involvement of lipid signaling from CD4^+^ or CD8^+^ T cells toward T1D development has not been examined. Herein, we investigated the impact of iDL signaling in CD4^+^ or CD8^+^ T cells on T1D development. Utilizing adoptive transfer, flow cytometry, qPCR, imaging, lipidomics, and ex-vivo approaches, we find that CD4^+^ and CD8^+^ T cells with reduced iPLA_2_β exhibit a mitigated inflammatory landscape and are less effective in inducing T1D. Our findings raise the possibility that targeting CD4^+^ and/or CD8^+^ T-cell–iDL signaling can be beneficial in mitigating T1D development.

## 2 Methods

### 2.1 Animal generation and monitoring

Wild-type NOD.*iPLA_2_β* ^+/+^ *(NOD*), NOD.*iPLA_2_β^+/-^ (NOD.HET*), and NOD.*iPLA_2_β^-/-^ (NOD.KO*) mice were generated and genotyped, as described (20). Littermates were maintained with free access to food and acid water (pH 3–4) according to the University of Alabama at Birmingham (UAB) Institutional Animal Care and Use Committee (IACUC) policies. Only female NOD mice, recognized to exhibit a higher diabetes incidence than male NOD littermates (Jax Lab, https://www.jax.org/jax-mice-and-services/strain-data-sheet-pages/diabetes-chart-001976), were used. Blood glucose levels, measured from tail vein blood samples (2 μL) with the Breeze 2 Blood Glucose Monitoring System (SKU:combo165, Bayer Healthcare, Mishawaka, IN), were recorded weekly. Diabetes incidence was recorded upon two consecutive blood glucose readings ≥275 mg/dL, at which time the mouse was euthanized in a CO_2_ chamber, as per UAB-IACUC guidelines. Mice that remained diabetes-free were euthanized at 30 weeks of age. At the time of euthanizing, blood was collected from the orbital sinus into BD Microtainer Tubes with serum separator for plasma (ELISA Kit, 90080; Crystal Chem, Elk Grove Village, IL) and lipidomics [by mass spectrometry (MS)] analyses, and the pancreas excised for analyses described in Section 2.6.

### 2.2 Splenocyte adoptive transfer

Spleens were excised from pre-diabetic 12-week-old *NOD*, NOD.*HET*, and NOD.*KO* mice and splenocytes prepared, as described (12). The cells from each genotype were pooled separately and resuspended in PBS. They were then administered (*i.p*., 2.5 × 10^6^ cells/mouse in 70 μL of PBS) to 4- to 5-week-old female immunodeficient NOD.*scid* (Jax Labs, #001976, Bar Harbor, ME) recipients.

### 2.3 mRNA analyses

Cells were centrifuged and lysed in 500 μL of Trizol (Invitrogen, #15596-026, Carlsbad, CA). Total RNA was prepared and purified using a GeneJET RNA purification kit (Thermo Fisher Scientific, #K0732, Waltham, MA). The RNA was converted to cDNA using the iScript cDNA Synthesis Kit (Bio-Rad, #1708891, Hercules, CA), and the cDNA transcripts were amplified, as described (3), with forward/reverse primers targeting iPLA_2_β (*Pla2g6*) or sEH (*Ephx2*) genes: *Pla2g6*, (5′-GAGATGGTCAAAGCCCTCATTG-3′)/(5′-TTGGAGGCTATCAATGCAGGAG-3′); *Ephx2*, (5′-GCGTTCGACCTTGACGGAG-3′/5′TGTAGCTTTCATCCATGAGTGGT-3′); *granzyme B*, (5′-ATCAAGGATCAGCAGCCTGA-3′/5′-TGATGTCATTGGAGAATGTCT-3′). The reverse transcription quantitative real-time polymerase chain reaction (RT-qPCR) analyses were then carried out using SYBR Select Mastermix (Invitrogen, #4472908, Carlsbad, CA), according to the manufacturer’s instructions, using *18S* as an internal control. Relative gene expression levels were determined using the 2^–ΔΔCt^ method.

### 2.4 T-cell adoptive transfer

Splenocytes were prepared from pre-diabetic 12-week-old mice, as above, and StemCell EasySep Mouse CD4 positive (catalog no. 18952) and StemCell EasySep Mouse CD8 negative (catalog no. 18952) selection kits were used to prepare separated populations of CD4^+^ and CD8^+^ T cells, according to manufacturer’s instructions. Purity of the CD4^+^ and CD8^+^ T-cell populations was verified by flow cytometry analyses using anti-CD4^+^ APC (BD Biosciences, #553051, San Diego, CA) and anti-CD8^+^ fluorescein isothiocyanate (FITC) (BD Biosciences, #553030, San Diego, CA) markers. Next, *iPLA_2_* β mRNA was analyzed by qPCR to confirm reduction in the NOD.*HET* T cells. The cells were then administered (*i.p*.) to 4- to 5-week-old recipient NOD.*scid* in a 3:1 ratio (7.5 × 10^6^ CD4^+^: 2.5 × 10^6^ CD8^+^), as described (27). The recipient mice were divided into four groups depending on the administered donor T cells combination: (1) *NOD* CD4^+^ T cells + *NOD* CD8^+^ T cells, (2) NOD.*HET* CD4^+^ T cells + *NOD* CD8^+^ T cells, (3) *NOD* CD4^+^ T cells + NOD.*HET* CD8^+^ T cells, and (4) NOD.*HET* CD4^+^ T cells + NOD.*HET* CD8^+^ T cells.

### 2.5 Glucose tolerance

Intraperitoneal glucose tolerance tests (IPGTTs) were performed, as described (12). Overnight-fasted mice were administered (i.p.) glucose 2 g/kg body weight in filter-sterilized acid H_2_O and tail vein samples (2 μL) collected over a 2 h–period for glucose measurements.

### 2.6 Insulitis, islet immunostaining, and microscopy

Paraffin sections (6 μm) of pancreata were prepared and stained with hematoxylin-eosin (H/E) for histological assessment of islet infiltration. Sections containing islets were then processed for immunostaining using an antigen retrieval protocol, as previously described (28). The sections were incubated overnight at 4°C with 1° antibody goat anti-insulin (1:100) (#15848-1-AP, ProteinTech, Rosemont, IL) with fluorescence-labeled 2° antibody FITC (1:50) (#F2765, Invitrogen, Carlsbad, CA) in the dark (1 h, room temperature). Nuclei were stained with DAPI for 3 min, and the ratio of total insulin-stained islet region to H/E–stained pancreas section was used to calculate β-cell area. Images were captured (4× and 40× magnification) and total islet and non-infiltrated areas (pixels) were determined on cellSens imaging software. Pancreas section and islet images were captured on an Olympus IX81 microscope using cellSens dimension software and analyzed using cellSens software (National Institutes of Health).

### 2.7 *Ex vivo* Th1 and CD8^+^ differentiation

T1D is recognized to be a Th1-driven disease with IFNγ production correlating with diabetes progression in NOD mice (29). The CD4^+^ T cells were therefore differentiated to Th1 cells and stimulated in Dulbecco’s Modified Eagle Medium (DMEM) with 10% FCS, 200 mM l-glutamine, 100 mM sodium pyruvate, 10 mM MEM non-essential amino acids, 10 mg/mL gentamicin, 1 M HEPES and 50 μM β-mercaptoethanol with plate-bound 10 μg/mL anti-CD3ϵ (BioXCell, BE0001-1, Lebanon, NH), and soluble 1 μg/mL anti-CD28 (eBioscience, #16-0281-82, Carlsbad, CA), 10 μg/mL anti–IL-4 (BioXCell, Cat #11B11), and 10 ng/mL IL-12 (R&D Systems, #419-ML-010/CF, Minneapolis, MN) for 72 h. Naïve CD8^+^ T cells were stimulated with DMEM, as described above, with plate-bound 10 μg/mL anti-CD3ϵ (BioXCell, BE0001-1, Lebanon, NH) and soluble 1 μg/mL anti-CD28 (eBioscience, #16-0281-82, Carlsbad, CA). Culturing of CD8^+^ cells was performed, as described (30). In some experiments, the cells were pretreated with either grapiprant, an EP4r antagonist (31), or TPPU, an inhibitor of soluble epoxide hydrolase (sEH) (32).

### 2.8 *Ex vivo* functional analyses

The CD4^+^ (1 × 10^6^/mL) and CD8^+^ (1 × 10^5^/mL) T cells were plated in DMEM media and incubated for 24 h at 37°. The CD8^+^ T cells were then exposed to 1-(4-trifluoro-methoxy-phenyl)-3-(1-propionylpiperidin-4-yl) urea (TPPU, 10 µmol/L), grapiprant (MedChem Express, #HY-16781, Monmouth, NJ, 1 µmol/L), or exogenous PGE_2_ (Cayman chemical, #14010, Ann Arbor, MI 1 µmol/L) alone or with the ebioscience stimulation cocktail (Ebioscience, #00-4970-03, Carlsbad, CA) for 4 h in the presence of brefeldin A (BioLegend, #420601, San Diego, CA) to prevent cytokine secretion. Cells were collected for flow cytometry and media for lipidomics analyses. At 72 h, the CD4^+^ T cells were exposed to the same treatments as CD8^+^ T cells and prepared for flow cytometry analysis. The media were collected for lipidomics.

### 2.9 Surface and intracellular staining

Cell surface staining was performed, separately, on single-cell suspensions of CD4^+^ and CD8^+^ T cells with anti-CD4^+^ APC (BD Biosciences, #553051, San Diego, CA) and anti-CD8 FITC (BD Biosciences, #553030, San Diego, CA), anti-CD45 BV605 (BioLegend, #103139, San Diego, CA). For the analysis of cytokine production, cells were stimulated with eBioscience stimulation cocktail for 24 h in the presence of brefeldin A and then stained using the Foxp3 Permeabilization/Fixation kit (Invitrogen, #00-5523-00, Carlsbad, CA). A viability dye (Invitrogen, #L34965, Carlsbad, CA) was applied to exclude dead cells. Intracellular flow cytometry was performed according to the manufacturer’s protocol, as displayed in **Supplementary Table S1**. Schemas of the flow cytometry gating are illustrated in **Supplementary Figure S1**.

### 2.10 Analysis of T-cell eicosanoids by mass spectrometry

The CD4^+^ T cell and CD8^+^ T cell prepared from mice were stimulated as above and the media processed for lipidomics analyses, as described (20, 33–41). Briefly, media (4 mL) were combined with an IS mixture composed of 10% methanol (400 μL), glacial acetic acid (20 μL), and internal standard (20 μL) containing the following deuterated eicosanoids (1.5 pmol/μL, 30 pmol total) purchased from Cayman Chemicals: (d4) 6-keto-prostaglandin F_1_α, (d4) prostaglandin F_2_α, (d4) prostaglandin E_2_, (d4) prostaglandin D_2_, (d8) 5-hydroxyeicosatetranoic acid (5-HETE), (d8) 12-hydroxyeicosatetranoic acid (12-HETE) (d8) 15-hydroxyeicosatetranoic acid (15-HETE), (d6) 20-hydroxyeicosatetranoic acid (20-HETE), (d11) 8,9-epoxyeicosatrienoic acid, (d8) 14,15-epoxyeicosatrienoic acid, (d8) arachidonic acid, (d5) eicosapentaenoic acid, (d5) docosahexaenoic acid, (d4) prostaglandin A_2_, (d4) leukotriene B_4_, (d4) leukotriene C_4_, (d4) leukotriene D_4_, (d4) leukotriene E_4_, (d5) 5(S),6(R)-lipoxin A_4_, (d11) 5-iPF_2_α-VI, (d4) 8-iso prostaglandin F_2_α, (d11) (±)14,15-DHET, (d11) (±)8,9-DHET, (d11) (±)11,12-DHET, (d4) prostaglandin E_1_, (d4) thromboxane B_2_, (d6) dihomo-γ linoleic acid, (d5) resolvin D2, (d5) resolvin D1 (RvD1), (d5) maresin2, (d7) 5-oxoETE, and (d5) resolvin D3. Samples and vial rinses (5% MeOH; 2 mL) were applied to Strata-X SPE columns (Phenomenex), previously washed with methanol (2 mL) and then dH_2_O (2 mL). Eicosanoids eluted with isopropanol (2 mL) were dried in vacuuo and reconstituted in EtOH:dH_2_O (50:50;100 μL) prior to ultra-high performance liquid chromatography electrospray ionization-MS/MS (UPLC ESI-MS/MS) analysis. Plasma lipidomics analyses were performed by combining plasma (200 μL) with 800 μL of LCMS H_2_O followed by the addition of an IS mixture composed of 10% methanol (100 mL), glacial acetic acid (5 mL), and internal standard (20 μL) containing the following deuterated eicosanoids (1.5 pmol/μL, 30 pmol total).

The eicosanoids were separated using a Shimadzu Nexera X2 LC-30AD coupled to a SIL-30AC auto injector, coupled to a DGU-20A5R degassing unit in the following way: A 14 min, reversed phase LC method utilizing an Acentis Express C18 column (150 mm × 2.1 mm, 2.7 µm) was used to separate the eicosanoids at a 0.5 ml/min flow rate at 40°C. The column was equilibrated with 100% Solvent A [acetonitrile:water:formic acid (20:80:0.02, v/v/v)] for 5 min and then 10 µL of sample was injected. One hundred percent Solvent A was used for the first 2 min of elution. Solvent B (acetonitrile:isopropanol:formic acid [20:80:0.02, v/v/v]) was increased in a linear gradient to 25% Solvent B at 3 min, to 30% at 6 min, to 55% at 6.1 min, to 70% at 10 min, to 100% at 10.10 min, 100% Solvent B was held constant until 13.0 min, where it was decreased to 0% Solvent B and 100% Solvent A from 13.0 min to 13.1 min. From 13.1 min to 14.0 min, Solvent A was held constant at 100%.

The eicosanoids were analyzed via MS using an AB Sciex Triple Quad 5500 Mass Spectrometer. Q1 and Q3 were set to detect distinctive precursor and product ion pairs. Ions were fragmented in Q2 using N2 gas for collisionally-induced dissociation. Analysis used multiple-reaction monitoring in negative-ion mode. Eicosanoids were monitored using precursor → product MRM pairs. The MS parameters used were Curtain Gas: 20 psi; CAD: Medium; Ion Spray Voltage: −4500 V; Temperature: 300°C; Gas 1: 40 psi; Gas 2: 60 psi; Declustering Potential, Collision Energy, and Cell Exit Potential vary per transition.

### 2.11 Statistical analyses

Significant differences in T1D incidence were determined by the Mantel-Cox test. For all other analyses, “p” values were determined using the Student’s t-test (for analyses between two groups) or one-way analysis of variance (ANOVA) followed by Tukey’s multiple comparisons (for analyses between multiple groups). For lipidomics analyses, “p” values were determined by two-way ANOVA, followed by Dunnett’s multiple comparisons test. All statistical analyses were performed using GraphPad Prism (version 9) and a “p” value less than 0.05 was defined as significant.

## 3 Results

### 3.1 Splenocyte iPLA_2_β expression in NOD mice

**Figure 1:**
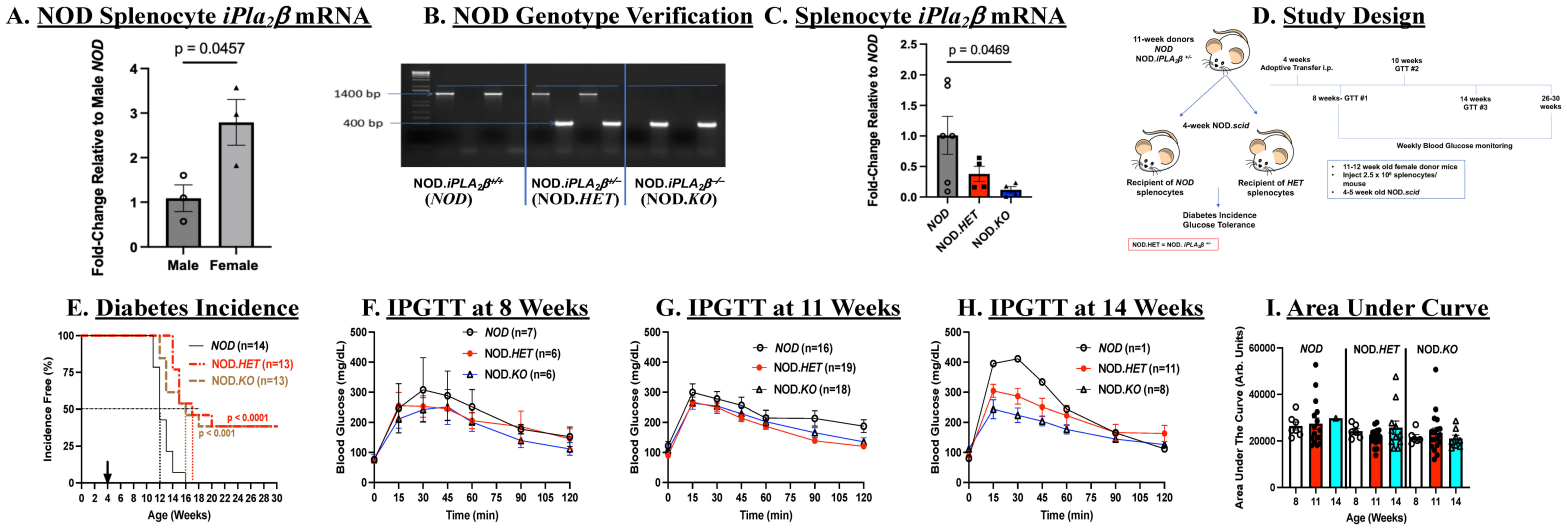
Splenocytes adoptive transfer. **(A)** Comparison of *iPla_2_β* mRNA in *NOD* males and females. Splenocytes were prepared from 8- to 10-week-old wild-type *NOD* mice and processed for qPCR analysis of *iPla_2_β* mRNA. Fold-change relative to male *NOD* are presented as mean ± SEM (*n* = 3 in each group). **(B)** Genotyping. DNA was generated from tail clips and progeny were genotyped by PCR analyses. Reactions were performed in the presence of primers for the *NOD* sequence or for the disrupted sequence (NOD.*iPLA_2_β^+/-^) for each mouse. Representative expected bands for the wild-type NOD* (1400 bp), NOD.*iPLA_2_* β^+/-^ (NOD.*HET*, 1400 and 400 bp), and NOD.*iPLA_2_* β^-/-^ (NOD.*KO*, 400 bp) (*n* = 2 in each group). **(C)** *iPla_2_β* mRNA in the NOD models. Splenocytes were prepared from 8- to 10-week-old female mice and processed for qPCR analyses. Fold-changes relative to *NOD* are presented as mean ± SEM (*NOD*, *n* = 6, NOD.*HET*, *n* = 4; NOD.*KO*, *n* = 5). **(D)** Experimental design. **(E)** Diabetes incidence. NOD.*scid* mice were administered splenocytes (*i.p*., 2.5 × 10^6^ cells/mouse in 70 μL of PBS) prepared from *NOD*, NOD.*HET*, and NOD.*KO*. Blood glucose was monitored weekly and T1D onset was recorded upon two consecutive readings >275 mg/dl. (*p*-values, *NOD* vs. NOD.*HET* and *NOD* vs. NOD.*KO* are indicated.) **(F–H)** Intraperitoneal glucose tolerance testing (IPGTT). Mice were fasted overnight before obtaining fasting blood glucose levels. The mice were then administered glucose 2 g/kg body weight and blood glucose in a 2 µL aliquot of tail vein blood was measured over a 2 h-period. **(I)** Area under the curve. Data obtained through the IPGTT were used to calculate the area under the curve, as an index of glucose tolerance.

We previously noted an intriguing direct relationship between islet *iPLA_2_β* expression and T1D susceptibility (12). Here, we find that *iPla_2_β* mRNA is threefold higher in splenocytes from pre-diabetic female NOD relative to age-matched male NOD (**Figure 1A**), raising the possibility that splenocyte-iDL signaling contributes to T1D development. To address this, NOD mice with reduced iPLA_2_β expression were generated, as described (20). Genotypes were verified by PCR analyses, which yielded expected band sizes of only 1400 bps for wild type (NOD.*iPLA_2_β* ^+/+^), 1400 and 400 bps for NOD.*iPLA_2_β* ^+/-^, and only 400 bps for NOD.*iPLA_2_β^-/-^* (**Figure 1B**); the mice were designated *NOD*, NOD.*HET*, and NOD.*KO*, respectively.

### 3.2 Transfer of splenocytes with reduced iPLA_2_β mitigates diabetes development

Immunodeficient and spontaneous diabetes-resistant NOD.*scid* mice develop diabetes with administration of splenocytes from *NOD* mice (42). We first examined if T1D incidence was affected with transfer of splenocytes with differential expression of iPLA_2_β. Prior to transfer, qPCR analyses confirmed decreases in *iPLA_2_β* mRNA in NOD.*HET* and NOD.*KO*, relative to *NOD*, splenocytes (**Figure 1C**). Female 4-week-old NOD.*scid* mice were administered splenocytes from *NOD*, NOD.*HET*, or NOD.*KO* donors, and monitored over a subsequent 26-week period (**Figure 1D**).

Weekly blood glucose measurements revealed a rapid T1D onset within 7 weeks post-transfer and 50% incidence by 8 weeks post-transfer in mice administered *NOD* splenocytes. By 12-weeks post-transfer, all mice in this group became diabetic (**Figure 1E**). In contrast, T1D onset was delayed by 1–3 weeks in mice administered NOD.*HET* or NOD.*KO* splenocytes, and 50% incidence was delayed 4 to 5 weeks. Even by 30 weeks of age, 40% of the mice in these two groups remained diabetes-free. The rapid onset of diabetes in the *NOD* cells recipients precluded meaningful comparisons of glucose tolerance after 11 weeks of age; however, IPGTTs (**Figures 1F–H**) performed in the remaining non-diabetic recipients suggested a trend (not significant) toward glucose intolerance between 8 and 14 weeks of age, as reflected by higher AUCs in recipients of cells (**Figure 1I**), inspite of weekly blood glucose levels being similar in all genotypes (**Supplementary Figure S2A**). In contrast, glucose tolerance remained steady during this period in the NOD.*HET* and NOD.*KO* recipients. These findings suggest that reduced iPLA_2_β expression in splenocytes delays T1D development, stabilizes glucose tolerance, and, most importantly, reduces T1D incidence.

### 3.3 Reduced insulitis and β-cell preservation following transfer of splenocytes with mitigated iDL signaling

**Figure 2:**
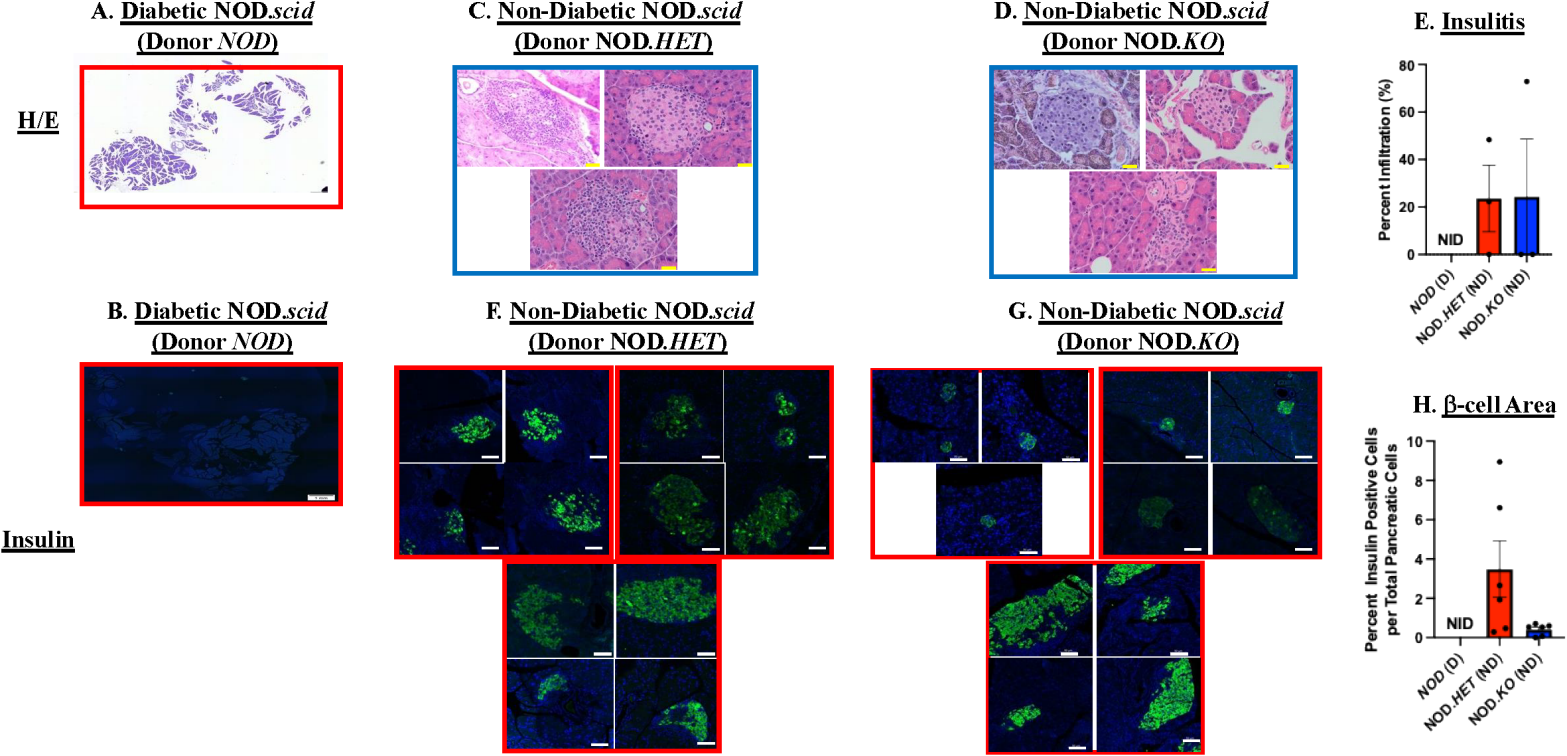
Islet infiltration and insulin staining. NOD mice were treated as described in **Figure 1**. **(A, C, D)** Hematoxylin/eosin (H/E) staining. Paraffin sections (6 μm) of pancreas were prepared (at onset of diabetes or at 30 weeks of age) from the *NOD*, NOD.*HET*, and NOD.*KO* groups and stained with H/E. Representative images from three mice in each group are presented. H/E-stained pancreas magnification is 4× and individual islet magnification is 40×. Scale bar of pancreas is 20 μm and islets scale bar is 50 μm. **(B, F, G)** Insulin staining. Paraffin sections (6 μm) were stained for insulin (green) and nucleus (DAPI, blue). Representative images of islets from three mice in each group are presented. Insulin-stained pancreas magnification is 4× and individual islet magnification is 40×. Scale bar for insulin-stained pancreas is 1 mm and individual islets 50 μm. **(E)** Insulitis. Percent infiltration for each islet was calculated as the value of noninfiltrated area subtracted from total islet area [% infiltrate = 100 × (total area–non-infiltrated area)/total area] using ImageJ software. Data are mean ± SEM of percent of islet infiltrated. *NOD*, NOD.*HET*, and NOD.*KO* groups were analyzed (*n* = 3 animals from each group; islets in each *NOD* = 0, NOD.*HET* = 4, 8, 10; and NOD.*KO* = 10, 2, 10. **(H)** β-cell area. Dividing the insulin-stained area, representing β cells, by total pancreas area: % β cells = 100 × [β-cell area sum per pancreas/pancreas area total]. Data are presented as mean ± SEM (*NOD* (*n* = 5), NOD.*HET* (*n* = 6), and NOD.*KO* (*n* = 6); islets in each *NOD* = 0 in each; NOD.*HET* = 1, 1, 2, 16, 37, and 2; and NOD.*KO* = 13, 20, 7, 25, 2 and 0. [**(E, H)** NID = no islets detected].

In view of the reduced diabetes incidence in the recipients of NOD.*HET* and NOD.*KO* cells, islet infiltration by H/E and β-cell area as reflected by insulin staining were assessed. Not surprisingly, there were no discernible islets (**Figure 2A**) or insulin-stained cells (**Figure 2B**) in pancreata from NOD.*scid* recipients at T1D onset. In comparison, islets could be identified in pancreas sections from the non-diabetic NOD.*scid* recipients of NOD.*HET* and NOD.*KO* cells at 30 weeks of age (**Figures 2C, D**). Moreover, while islets from these mice exhibited mild (∼20%) infiltration (**Figure 2E**), there was evidence of insulin staining in islets from both groups (**Figures 2F–H**), suggesting a preservation of sufficient β-cell mass to sustain normoglycemia.

**Figure 3:**
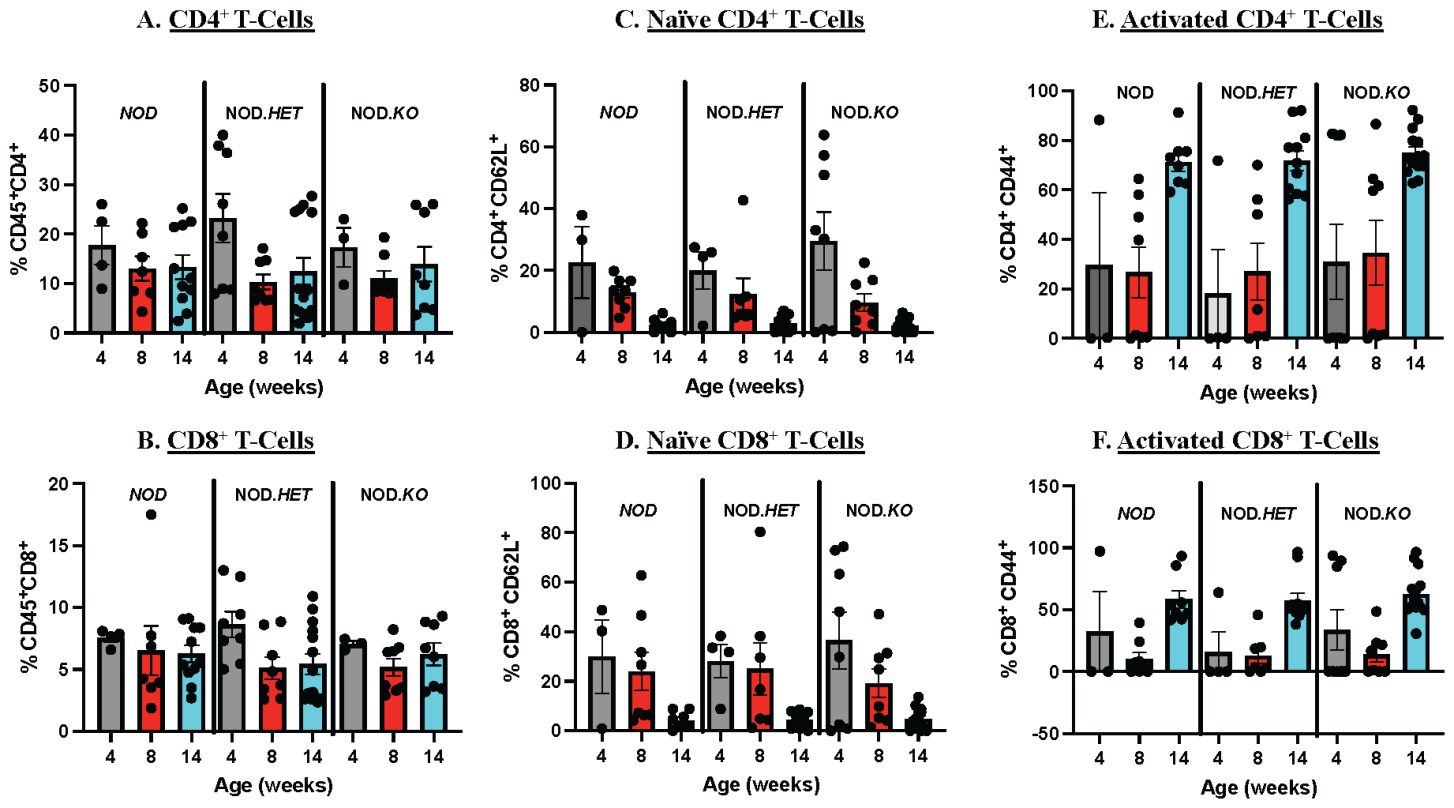
Splenocyte Immune Cell Composition. Age-matched *NOD*, NOD.*HET*, and NOD.*KO* female spleen were processed for flow cytometry to analyze the splenic immune cell composition. Analyses were done at 4, 8, and 14 weeks. **(A)** CD4^+^ T cells relative to CD45^+^. **(C)** Naïve CD4^+^ T cells relative to total CD4^+^ population. **(E)** Activated CD4^+^ T cells relative to total CD4^+^ population. **(B)** CD8^+^ T cells relative to CD45^+^. **(D)** Naïve CD8^+^ T cells relative to total CD8^+^ population. **(F)** Activated CD8^+^ T cells relative to total CD8^+^ population. Surface markers utilized included CD45, CD4, CD8, CD62L, and CD44. *NOD:* 4 weeks (*n* = 4), 8 weeks (*n* = 7), and 14 weeks (*n* = 11). NOD.*HET*: 4 weeks (*n* = 8), 8 weeks (*n* = 8), and 14 weeks (*n* = 14). NOD.*KO*: 4 weeks (*n* = 3), 8 weeks (*n* = 8), and 14 weeks (*n* = 8).

To determine if the reduced diabetes outcomes were due to differences in temporal abundances of splenic T cells, we performed flow analyses. We found that the frequencies of CD4^+^ and CD8^+^ T cells relative to CD45^+^ cells (**Figures 3A, D**), and of naive (**Figures 3B, E**) or activated (**Figures 3C, F**) CD4^+^ and CD8^+^ T cells at 4, 8, and 14 weeks of age were similar between the *NOD*, NOD.*HET*, and NOD.*KO*.

These findings suggest that the reduced T1D incidence with decreased splenocyte-iDL signaling is associated with mitigated insulitis and preservation of β-cells and is independent of differences in splenic CD4^+^ or CD8^+^ T-cell abundances and activation.

### 3.4 Transfer of T cells with reduced iPLA_2_β mitigates diabetes development

**Figure 4:**
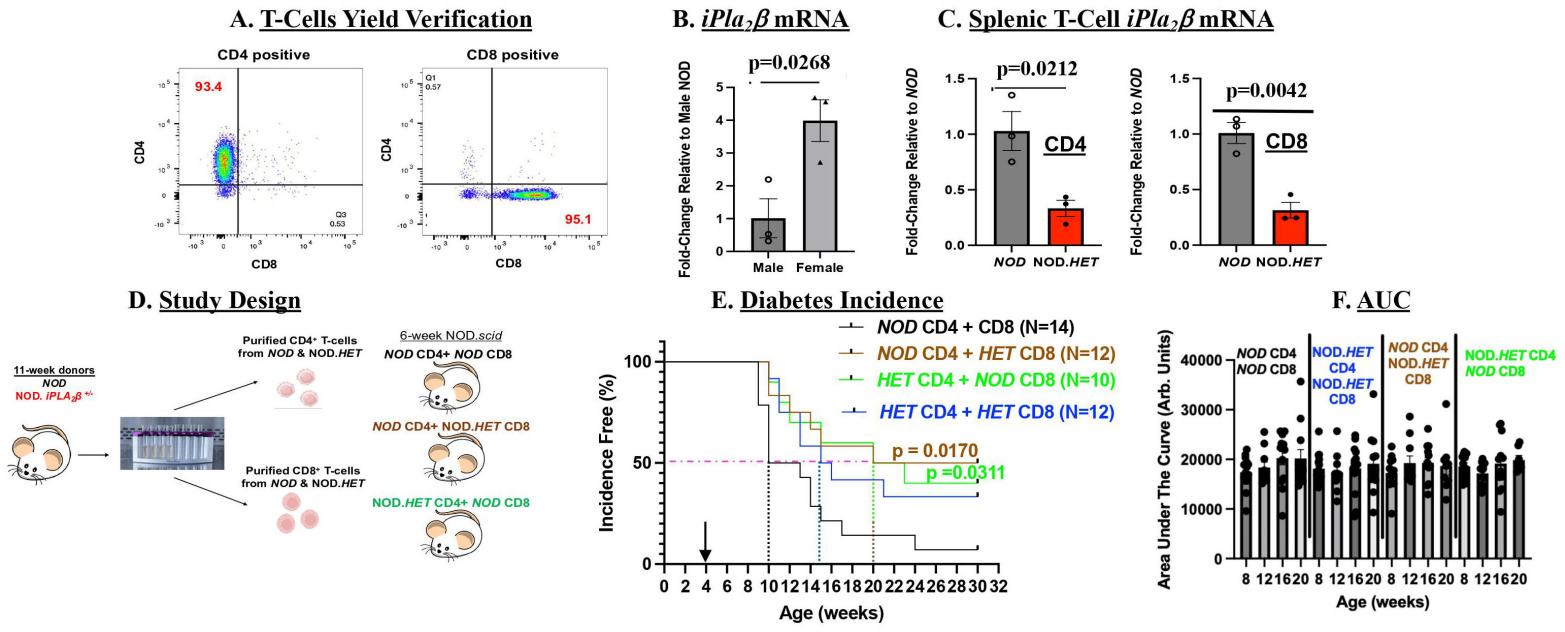
T-cell adoptive transfer. **(A)** Verification of T-cell purity. Splenic T cells were prepared using StemCell positive and negative selection columns and purity assessed by flow analyses using markers for CD4^+^ and CD8^+^ cells (**Supplementary Figure S1**). CD4^+^ cells and CD8^+^ T cells are represented in quadrants 1 and 3, respectively. **(B)** *iPla_2_* β mRNA. Purified T cells 10- to 12-week-old from male and female NOD were processed for qPCR analyses. Fold-change relative to *NOD* are presented as mean ± SEM (*p*-value of male vs. female indicated, *n* = 3 in each group). **(C)** Splenic T-cell–*iPla_2_* β mRNA. Purified T cells were processed for qPCR analyses. Fold-changes relative to *NOD* are presented as mean ± SEM (*p*-values of *NOD* vs. NOD.*HET* indicated, *n* = 3 in each group). **(D)** Experimental design. **(E)** Diabetes incidence. NOD.*scid* (5-week-old) were administered (*i.p*.) CD4^+^/CD8^+^ T cells in a 3:1 ratio (7.5 × 10^6^ CD4^+^: 2.5 × 10^6^ CD8^+^) and blood glucose was monitored weekly and T1D onset recorded, as described in **Figure 1**. (*p*-values of *NOD* CD4 + *HET* CD8 or *HET* CD4 + *NOD* CD8 *vs*. *NOD* CD4 + CD8 are indicated.) **(F)** Area under the curve. Data obtained through the IPGTT were used to calculate the area under the curve, as an index of glucose tolerance.

As mouse splenocytes are enriched in CD4^+^ and CD8^+^ T cells (43), we prepared splenic CD4^+^ and CD8^+^ T cells to assess the specific impact of iDL signaling from these cells on T1D development. Given the similar outcomes with NOD.*HET* and NOD.*KO* splenocytes and to reduce breeding burden, the *NOD* and NOD.*HET* were chosen for subsequent analyses. Sequential positive and negative selection columns were used to isolate the CD4^+^ and CD8^+^ T cells, respectively, from spleens and purity verified by flow cytometry was 93%–95% (**Figure 4A**). Consistent with the splenocytes, *iPla_2_β* mRNA in the CD4^+^ and CD8^+^ T-cell pool was significantly higher in the females than males (**Figure 4B**). Furthermore, qPCR analyses confirmed decreases in *iPla_2_β* mRNA in both CD4^+^ and CD8^+^ T cells from female NOD.*HET*, relative to *NOD* cells (**Figure 4C**).

In initial studies, NOD.*scid* mice were administered CD4^+^ and CD8^+^ T cells purified from *NOD* or NOD.*HET* mice (**Figure 4D**). As with splenocytes transfer, *NOD* T cells induced a rapid (by 5-week post-transfer) diabetes onset with 50% incidence reached by 6-week post-transfer and near 100% incidence by 20 weeks post-transfer (**Figure 4E**). In contrast, administration of NOD.*HET* cells delayed onset by only 1 week; however, 50% incidence was delayed 5 weeks and 40% of recipients in this group remained diabetes-free through 30 weeks of age (p = 0.0657). These finding suggested contribution of T-cell iDL signaling toward diabetes development.

To assess the specific roles of CD4^+^ and CD8^+^ T-cell iDL signaling, NOD.*scid* mice were next transferred mixture of CD4^+^ and CD8^+^ T cells from *NOD* and NOD.*HET* and monitored for 30 weeks (**Figure 4D**). In comparison to recipients of *NOD* CD4^+^ + *NOD* CD8^+^ T cells, in mice administered *NOD* CD4^+^ + NOD.*HET* CD8^+^ T cells or *NOD* CD8^+^ + NOD.*HET* CD4^+^ T cells, T1D onset was delayed by one week and 50% incidence 10 weeks. Overall, the incidence in these two groups remained between 40% and 50% through the end of the 30-week monitoring period (**Figure 4E**). Blood glucose levels groups (**Supplementary Figure S2B**) and glucose tolerance (**Figure 4F**) in the non-diabetic mice among the four groups were found to be similar. These data suggest that iDL signaling from CD4^+^ or CD8^+^ T cells can impact T1D development.

### 3.5 Reduced insulitis and β-cell preservation in NOD.*scid* recipients of T cells with mitigated iDL signaling

**Figure 5:**
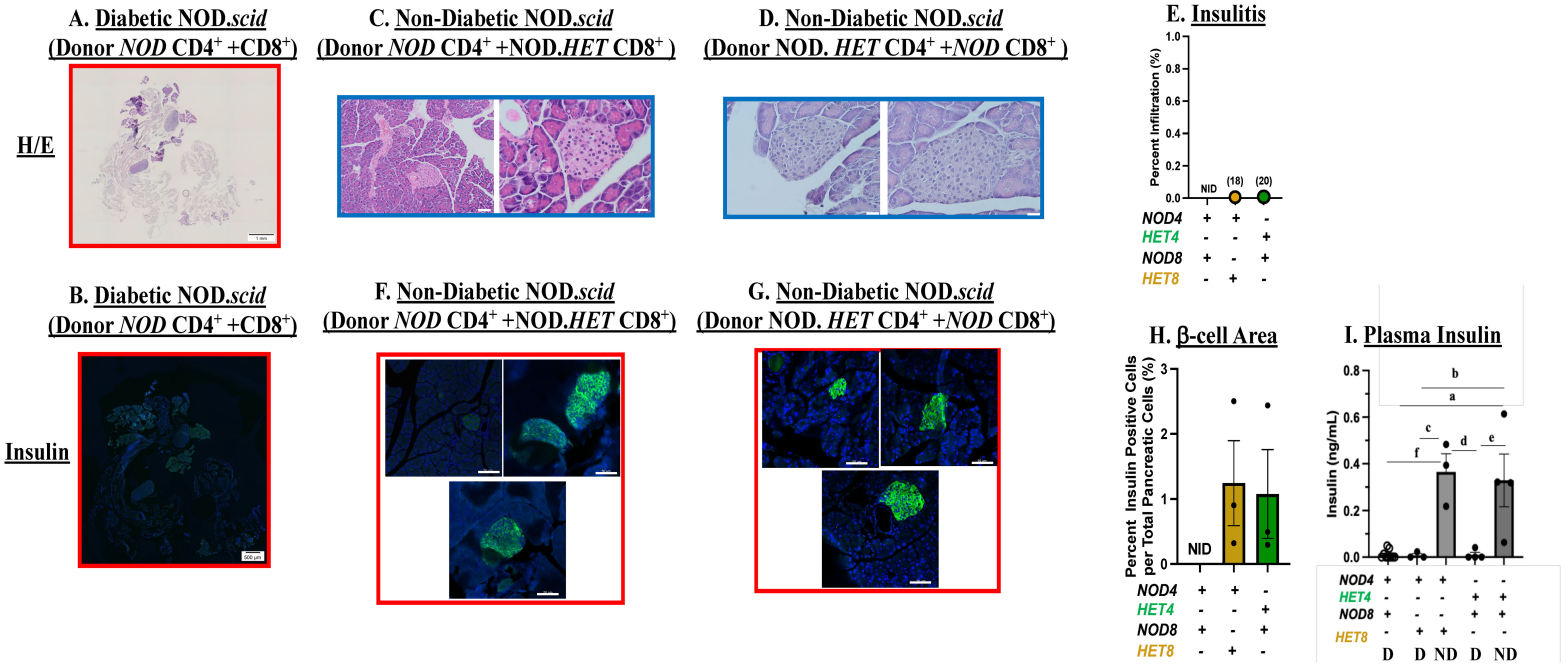
Islet infiltration and insulin staining. Mice were treated as described in **Figure 3**. **(A, C, D)** Hematoxylin/eosin (H/E) staining. Paraffin sections (6 μm) of pancreas were prepared (at T1D onset or at 30 weeks of age) from NOD.*scid* recipients of *NOD* CD4^+^ T cells + *NOD* CD8^+^, *NOD* CD4^+^ + NOD.*HET* CD8^+^ or NOD.*HET* CD4^+^ + *NOD* CD8^+^ T cells and stained with H/E. Representative images from three mice in each group are presented. H/E-stained pancreas magnification is 4× and individual islet magnification is 40×. Scale bar of H/E-stained pancreas is 1 mm and individual islets are 50 μm. **(B, F, G)** Insulin staining. Paraffin sections (6 μm) of pancreas were stained for insulin (green) and nucleus (DAPI, blue). Representative images of islets from three mice in each group are presented. Insulin-stained pancreas magnification is 4× and individual islet magnification is 40×. Scale bar of insulin-stained pancreas is 500 μm and individual islets are 50 μm. **(E)** Insulitis. Percent infiltration for each islet was calculated as the value of non-infiltrated area subtracted from total islet area [% infiltrate = 100 × (total area − non-infiltrated area)/total area] using ImageJ software. Data are presented as mean ± SEM (three animals in each group; islets in each *NOD* CD4^+^ *+ NOD* CD8^+^ = 0 in each; NOD.*HET* CD4^+^ *+ NOD* CD8^+^ = 10, 1, and 7 (*n* = 18); and *NOD* CD4^+^ *+* NOD.*HET* CD8^+^ = 8, 2, and 10 (*n* = 20). **(H)** β-cell area. Determined by dividing the insulin-stained area, representing β cells, by total pancreas area: % β cells = 100 × (β-cell area sum per pancreas/pancreas area total). Data are presented as mean ± SEM (*n* = 3 animals in each group; islets in each *NOD* CD4^+^ *+ NOD* CD8^+^= 0 in each; NOD.*HET* CD4^+^ *+ NOD* CD8^+^ = 10, 1, and 7, and *NOD* CD4^+^ *+* NOD.*HET* CD8^+^ = 8, 2, and 10. (E and H, NID = no islets detected). **(I)** Circulating Insulin levels. Plasma was collected from diabetic **(D)** *NOD* CD4^+^ + *NOD* CD8^+^ (*n* = 9), *NOD* CD4^+^ + NOD.*HET* CD8^+^ (*n* = 3), NOD.*HET* CD4^+^ + *NOD* CD8^+^ (*n* = 4) and non-diabetic (ND) *NOD* CD4^+^ + NOD.*HET* CD8^+^ (*n* = 3), NOD.*HET* CD4^+^ + *NOD* CD8^+^ (*n* = 4) mice and circulating insulin levels were determined by ELISA. (^a^ *NOD* CD4^+^ + *NOD* CD8^+^ D vs. NOD.*HET* CD4^+^ *+ NOD* CD8^+^ ND, *p* < 0.005; ^b^ *NOD* CD4^+^ + NOD.*HET* CD8^+^ D vs. NOD.*HET* CD4^+^ + *NOD* CD8^+^ ND, *p* < 0.01; ^c^ *NOD* CD4^+^ + NOD.*HET* CD8^+^ D vs. *NOD* CD4^+^ + NOD.*HET* CD8^+^ ND, *p* < 0.005; ^d^ *NOD* CD4^+^ + NOD.*HET* CD8^+^ ND vs. NOD.*HET* CD4^+^ + *NOD* CD8^+^ D, *p* < 0.005; ^e^NOD.*HET* CD4^+^ + *NOD* CD8^+^ D vs. NOD.*HET* CD4^+^ + *NOD* CD8^+^ ND, *p* < 0.005; and ^f^ *NOD* CD4^+^ + *NOD* CD8^+^ D vs. *NOD* CD4^+^ + NOD.*HET* CD8^+^ ND, *p* < 0.005).

We next assessed the impact of transferring CD4^+^ and CD8^+^ T cells with reduced iPLA_2_β on islet infiltration and β-cell area. As with the splenocytes transfer, following administration of *NOD* CD4^+^+CD8^+^ T cells no obvious islets (**Figure 5A**) or insulin staining (**Figure 5B**) could be detected in the NOD.*scid* recipient pancreata. In contrast, islets were evident in the pancreata of non-diabetic NOD.*scid* following administration of *NOD* CD4^+^ + NOD.*HET* CD8^+^ T cells (**Figure 5C**) or NOD.*HET* CD4^+^ + *NOD* CD8^+^ T cells (**Figure 5D**) and they were devoid of infiltration (**Figure 5E**). Furthermore, notable insulin staining (**Figures 5F, G**), reflecting preservation of β-cell mass (**Figure 5H**) was detected in the islets from these mice. Consistently, circulating insulin levels were significantly higher in these groups (**Figure 5I**), relative to diabetic NOD.*scid* in all groups. These findings suggest that iDL signaling from CD4^+^ or CD8^+^ T cells reduces insulitis and preserves β-cell mass and insulin content.

### 3.6 Impact of T-cell function with inhibition of select lipid signaling

**Figure 6:**
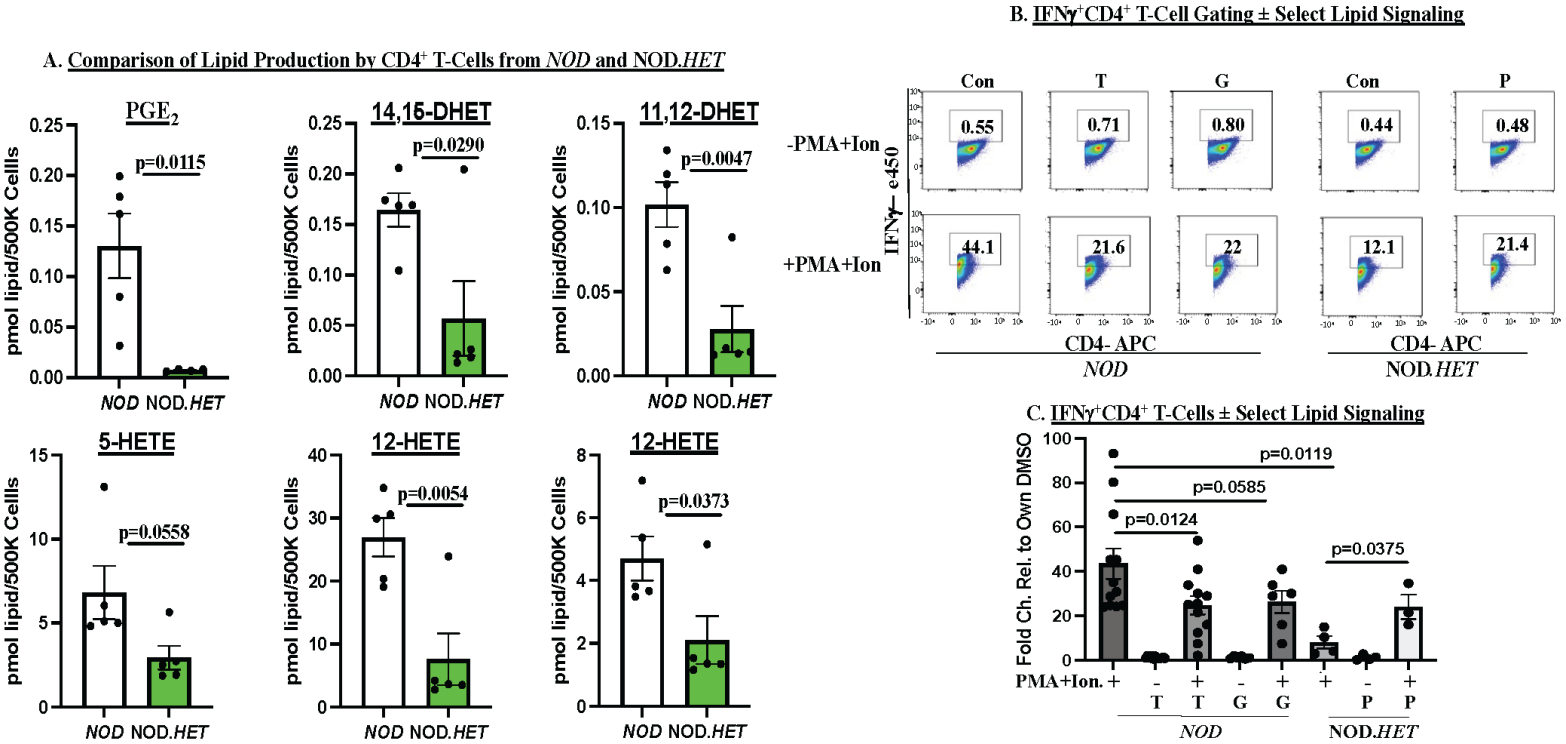
*Ex vivo* analyses of CD4^+^ T cells. **(A)** Lipidomics. CD4^+^ T cells were purified from 14-week-old *NOD* and NOD.*HET* and cultured for 48 h under basal conditions. The media was then collected for lipidomics analyses. (*p*-values, *NOD* vs. NOD.*HET* indicated.) **(B, C)** IFNγ production by CD4^+^ T cells. CD4^+^ T cells from *NOD* were differentiated to Th1 cells and cultured for 24 h. They were then pretreated with DMSO alone (Con), grapiprant (G, 1 μM), or TPPU (T, 10 μM) for 2 h prior to stimulation with ionomycin (670 μM) + PMA (40.5 μM) for an additional 24 h. The cells were then subjected to flow analyses to quantitate IFNγ^+^CD4^+^ T cells. The CD4^+^ T cells from NOD.*HET* were similarly differentiated and subsequently stimulated in the absence or presence of PGE_2_ (P, 1 μM). **(B)** Flow analyses demonstrating gating on IFNγ^+^CD4^+^ T cells under each condition. **(C)** Fold-changes in IFNγ^+^CD4^+^ T cells relative to DMSO are presented as mean ± SEM (*NOD, n* = 12; T, *n* = 12; *NOD* + T, *n* = 12; G (*n* = 6); *NOD* + G, *n* = 6; NOD.*HET*, *n* = 4; NOD.*HET* + P, *n* = 4; and NOD.*HET* + P + stimulation, *n* = 3. (*p*-values between groups indicated).

As the above observations suggested participation of CD4^+^ and CD8^+^ T-cell–iDL signaling in T1D development, we next assessed iDL production by CD4^+^ and CD8^+^ T cells. We found that the production of several proinflammatory lipids is higher in CD4^+^ T cells from the *NOD* than cells from NOD.*HET*. Among them were PGE_2_, DHETs, and HETEs (**Figure 6A**). All other eicosanoids production was similar between the two groups (data not shown). Inhibition of sEH has been reported to preserve inflammation-resolving epoxy fatty acids (EETs) (44). We therefore targeted PGE_2_ signaling and sEH-mediated conversion of EETs to DHETs (45). To address this, CD4^+^ T cells from *NOD* mice were stimulated in the absence (Control) and presence of an EP_4_ receptor antagonist (Grapiprant) to block PGE_2_ signaling or an inhibitor (TPPU) of sEH. Both interventions resulted in decreased stimulated expression of IFNγ in CD4^+^ T cells from NOD (**Figures 6B, C**), suggesting that both PGE_2_ and sEH pathways contribute to IFNγ production by CD4^+^ T cells. We further found that the stimulated expression of IFNγ in CD4^+^ T cells from NOD.*HET* is significantly reduced, in comparison with CD4^+^ T cells from *NOD*, and is rescued by supplementation of the media with PGE_2_. These findings identify impact of select iDL-related signaling on CD4^+^ T-cell effector function.

**Figure 7:**
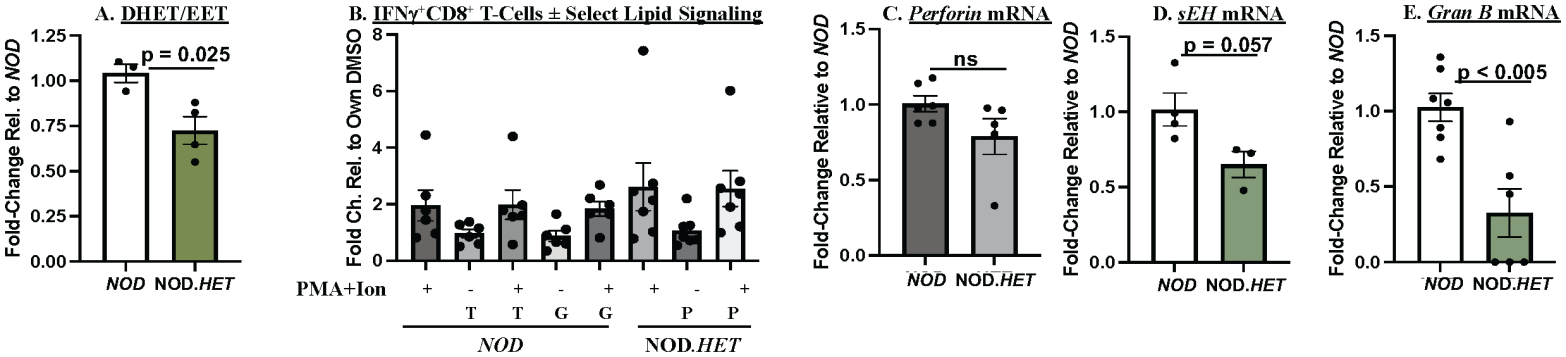
*Ex vivo* analyses of CD8^+^ T cells. **(A)** Lipidomics. CD8^+^ T cells were purified from 14-week-old *NOD* and NOD.*HET* and cultured for 24 h in the presence of hIL-2 (30 U/mL) and IL-7 (0.50 ng/mL). The cells were then stimulated using ebioscience stimulation cocktail containing ionomycin and PMA for 4 h. The cells were then collected for flow and media for lipidomics analyses. (*p*-value, *NOD* vs. NOD.*HET* indicated.) **(B)** IFNγ^+^CD8^+^ analyses. CD8^+^ T cells were treated with DMSO alone, grapiprant (G, 1 μM), or TPPU (T, 10 μM) in combination with ionomycin (670 μM) + PMA (40.5 μM) for 4 h. NOD.*HET* were similarly stimulated in the absence or presence of PGE_2_ (P, 1 μM). Fold changes in IFNγ^+^CD8^+^ T cells relative to DMSO are presented as mean ± SEM. (*NOD, n* = 6; T, *n* = 6; *NOD* + T, *n* = 6; G (*n* = 6); *NOD* + G, *n* = 6; NOD.*HET*, *n* = 7; NOD.*HET* + P, *n* = 7; and NOD.*HET* + P + stimulation, *n* = 7). **(C–E)** CD8^+^ T-cell mRNA analyses. Purified CD8^+^ T cells were processed for qPCR analyses of *perforin, sEh, and granzyme B* mRNA. Fold changes relative to *NOD* are presented as mean ± SEM (*NOD*, *n* = 3–7; NOD.*HET*, *n* = 3–6). (*p*-values, *NOD* vs. NOD.*HET* indicated).

In contrast to the CD4^+^ T cells, only the ratio of DHETs/EETs was lower in the NOD.*HET* CD8^+^ T cells, relative to the *NOD* (**Figure 7A**), but this was not associated with differences in cytokine production (**Figure 7B**) or perforin expression (**Figure 7C**). However, *sEh* (**Figure 7D**) and *granzyme B* (**Figure 7E**) mRNAs were decreased in the NOD.*HET*, relative to *NOD*, CD8^+^ T cells. These findings suggest an impact of select iDL signaling on sEH and granzyme B expression in the CD8^+^ T cells.

## 4 Discussion

Cytokine-mediated β-cell death occurs in an iPLA_2_β-dependent manner (17) and global inhibition or reduction of iPLA_2_β mitigates T1D development (12, 20). Those observations suggested that iDL signaling contributes to T1D development. T1D is a consequence of autoimmune destruction of β-cells by T cells (4, 46) and proinflammatory eicosanoids can have profound immunoregulatory and inflammatory effects in T cells (47); however, the impact of T-cell–derived lipids on T1D development has not been investigated. To address this, we utilized genetically modified NOD mice, an autoimmune model of spontaneous diabetes development, with reduced expression of iPLA_2_β (20).

To gain an insight into the potential role of T-cell–iDL signaling, splenocytes prepared from pre-diabetic *NOD* (wild type), NOD.*iPLA_2_β* ^+/-^ (NOD.*HET*), and NOD.*iPLA_2_β* ^-/-^ (NOD.*KO*) were transferred to immunodeficient NOD.*scid* mice. Not surprisingly, T1D onset was rapid and 100% in mice administered NOD splenocytes and the pancreata from these mice presented no evidence of discernible islets or insulin-positive β-cells. In contrast, T1D onset was delayed by 4 weeks in the NOD.*HET* and NOD.*KO* and 40% remained diabetes-free through 30 weeks of age. Importantly, islets from these mice exhibited only mild infiltration and contained insulin-positive β-cells at the end of the study period. These findings support a role for splenocyte-iDL signaling in T1D development. Moreover, the similarity in the incidence profiles in recipients of NOD.*HET* or NOD.*KO* cells suggests that a decrease in iPLA_2_β is sufficient to affect a reduction in T1D incidence. Several immune cell types comprise the splenocytes with T cells making up ~35% of the total cell population. The absence of changes in the abundances of CD4^+^ or CD8^+^ T cells in splenocytes with reduced iPLA_2_β expression raised the possibility that iDLs affect T-cell effector function. We therefore sought to identify the impact of iDL signaling specifically in CD4^+^ and CD8^+^ T cells on T1D development.

In NOD mice with a selective modification of iPLA_2_β in only the T cells, CD4^+^ and CD8^+^ T cells were purified from pre-diabetic *NOD* and NOD.*HET* spleens and were administered to NOD.*scid* mice, in a ratio known to induce diabetes (27, 48). As with the splenocyte transfer, *NOD* T cells induced a rapid T1D onset, 50% incidence within 6 weeks of transfer, near 100% incidence by 20-week post-transfer, and no readily identifiable islets in the pancreas. The remarkable similarities in the temporal profiles and overall incidences following either splenocyte or T-cell transfers from NOD donors confirm a predominant role of the CD4^+^ and CD8^+^ T-cell pools in splenocytes in inducing T1D. Intriguingly, onset was delayed by 1 week and 50% incidence by 5 weeks, and 40%–50% of the mice remained diabetes free with administration of NOD.*HET* T cells. These findings revealed for the first time the potential involvement of T-cell iDL signaling in T1D development, motivating further investigation of the impact of iDL signaling in CD4^+^ versus CD8^+^ T cells.

The iPLA_2_β manifests roles in multiple biological process, including apoptosis, signaling, cell proliferation, and homeostasis (49, 50). Furthermore, iPLA_2_β appears to differentially impact stress pathways leading to β-cell apoptosis (14, 16–19, 51–53). Our preliminary studies also suggested that diabetes incidence in NOD mice with global iPLA_2_β deficiency is similar to wild-type *NOD*, likely reflecting the absence of membrane lipid remodeling function of iPLA_2_β. In contrast, we found administration of a reversible inhibitor of iPLA_2_β (12, 20) or genetic reduction of iPLA_2_β significantly reduced T1D incidence (20) in the NOD. These findings suggested that reduced levels of iPLA_2_β can still manifest homeostatic functions while also promoting mitigation of inflammatory and autoimmune responses that contribute to T1D development. For these reasons and to reduce breeding burden, we utilized T cells with reduced iDL signaling for more detailed analyses. Employing a strategy of transferring different combinations of T cells from *NOD* and NOD.*HET*, we found only a modest 1 week delay in T1D onset in mice administered *NOD* CD4^+^+NOD.*HET* CD8^+^ or *NOD* CD8^+^+NOD.*HET* CD4^+^ T cells. However, 50% incidence in both groups was not reached until 20 weeks post-transfer and nearly half of the mice remained diabetes-free. Moreover, islets from these mice exhibited minimal infiltration and contained insulin-positive β-cells. These findings suggest that reduction in iDL signaling in either CD4^+^ or CD8^+^ T cells can lower T1D incidence, without affecting abundances of overall, naïve or activated T cells.

As such, we next addressed if the reduction in T1D incidence was accompanied by alterations in the production of iDLs by CD4^+^ or CD8^+^ T cells. Using MS approaches, we found that the production of only select lipids that included PGE_2_ and DHETs was increased in CD4^+^ T cells from the *NOD*, relative to NOD.*HET*. The lack of universal changes in all eicosanoids suggests targeted impact of the iPLA_2_β modification on select lipid-generating pathways, likely those that are involved during disease progression. Among the prostaglandins, PGE_2_ is recognized to be a potent inflammatory lipid (54, 55), and recent studies suggest that inhibition of sEH, via reducing inflammatory diols (i.e., DHETs) and/or stabilizing anti-inflammatory epoxy fatty acids (i.e., EETs), is effective in resolving inflammation (6, 44, 56). The EETs are reported to impact PGE_2_ signaling by downregulating PGE_2_ synthase, COX-2, and EP_4_r (57, 58) at the transcriptional level (6, 56, 59, 60). Consistent with these lipidomics findings, intervention of PGE_2_ and DHET signaling in *NOD* decreased IFNγ^+^ CD4^+^ T cells and supplementation of PGE_2_ restored IFNγ^+^CD4^+^ T-cell abundance in the NOD.*HET*. These findings are interpreted as specific involvement of iPLA_2_β-modulated PGE_2_-EP_4_r and EpFA-diol lipid signaling pathways in triggering effector functions of the CD4^+^ T cells.

In contrast to the CD4^+^ T cells, only a greater production of DHETs, accompanied by higher sEH expression, was evident in the *NOD* CD8^+^ T cells, relative to NOD.*HET* cells. However, these were not associated with differences in cytokine production or perforin expression between the two genotypes. However, granzyme B expression was significantly lower in the NOD.*HET*, relative to *NOD*, CD8^+^ T cells. These findings raise the possibility that the expression of granzyme B, which directly induces β-cell death, is modulated through sEH, which degrades inflammation-resolving EETs to proinflammatory DHETs (61–70). Alternatively, as CD8^+^ T cells are a major component of islet infiltrate during T1D development in rodents and humans (71–73) and an “exhausted” CD8^+^ T-cell phenotype is associated with slower disease progression and preservation of β-cells (74, 75), it might be speculated that select iDL signaling additionally facilitates cytotoxic CD8^+^ T-cell function.

iDL signaling is associated with β-cell apoptosis (14, 16–19, 51, 52), diabetic complications (76–78), and diabetes incidence (12, 20), suggesting a link between iPLA_2_β and T1D pathogenesis. It is well recognized that the autoimmune destruction of β-cells leading to T1D is a consequence of concerted signaling from β-cells, macrophages, and T cells (79). Of note, the protection against diabetes seemed to level off around 50% with transfer of CD4^+^ and CD8^+^ T cells with reduced iPLA_2_β. This is likely due to the strong autoimmune impact of transferred T cells in NOD.*scid* mice. Alternatively, we find that reductions in NOD macrophage-iDL signaling decrease activation of CD4^+^ and CD8^+^ T cells (*in revision*), and this is precluded in the NOD.*scid* with defective macrophages.

**Figure 8:**
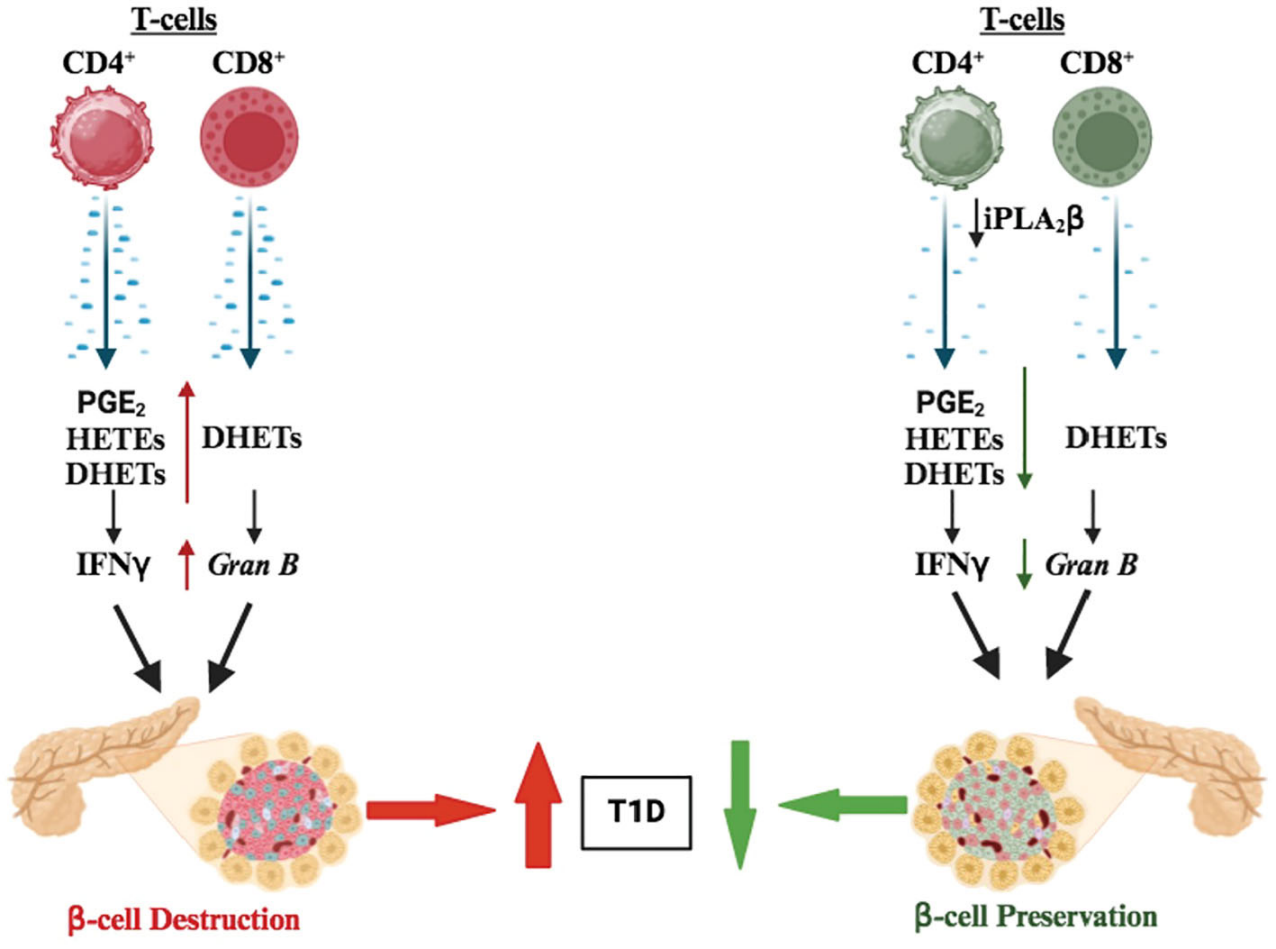
Proposed model of CD4^+^ and CD8 ^+^ T-cell–iPLA_2_β in T1D development. We suggest that iPLA_2_β in T cells promotes the production of inflammatory lipids (i.e., prostaglandins, DHETs, and leukotrienes), secretion of proinflammatory cytokines (i.e., IFNγ and TNFα), and *granzyme B* expression to promote T1D development. However, these outcomes are mitigated with reduced iPLA_2_β resulting in lowering T1D onset.

In summary, the current study offers forward-going insights into the participation of CD4^+^ and CD8^+^ T-cell–derived iDL signaling in T1D development (**Figure 8**). We demonstrate that select differential iDL signaling from these cells induces cytokine production from CD4^+^ T cells and granzyme B in CD8^+^ T cells, which are integral to immune processes that lead to β-cell death leading to T1D. In support, we find that reduction of select iDL signaling in CD4^+^ or CD8^+^ T cells mitigates an inflammatory landscape, reduces insulitis and preserves β-cell mass resulting in a delay in T1D onset and an overall decrease in T1D incidence.

The findings here complement our previous report that identified a similar lipid signature in normoglycemic children that were at high-risk for developing diabetes (20). A rapidly emerging concept, based on our studies, is that lipid signaling plays critical roles in inducing, propagating, and amplifying the immune responses. Yet, studies assessing the possibility of interfering with lipid signaling, in the context of immunotherapy, to alter the course of T1D development are missing. Presently, chimeric antigen receptor (CAR) T-cell therapy is being used to target and kill cancer cells. Our findings raise the possibility that similar strategies can be used to generate therapeutics that consider T cells with modified select lipid signaling to prevent or delay T1D onset.

## Data Availability

The raw data supporting the conclusions of this article will be made available by the authors, without undue reservation.
