# Supplementary material for: Differential lipid signaling from CD4+ and CD8+ T cells contributes to type 1 diabetes development
Source: Front Immunol. 2024 Sep 18;15:1444639. doi: 10.3389/fimmu.2024.1444639 (PMC11445035; doi:10.3389/fimmu.2024.1444639)
Supplement: Supplementary file 1 [file Presentation1.pptx]

## Slide 1
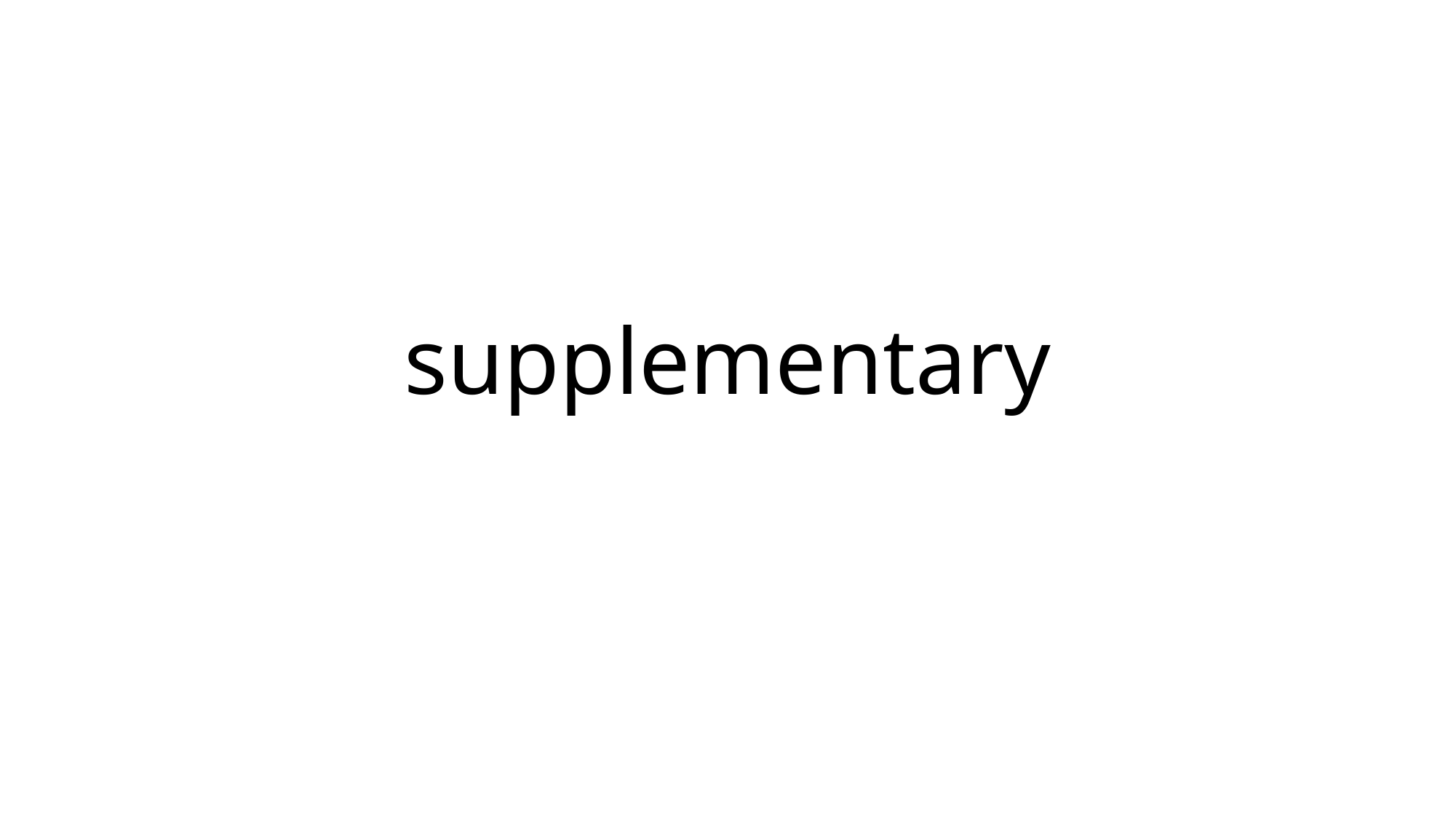

# supplementary

## Slide 2
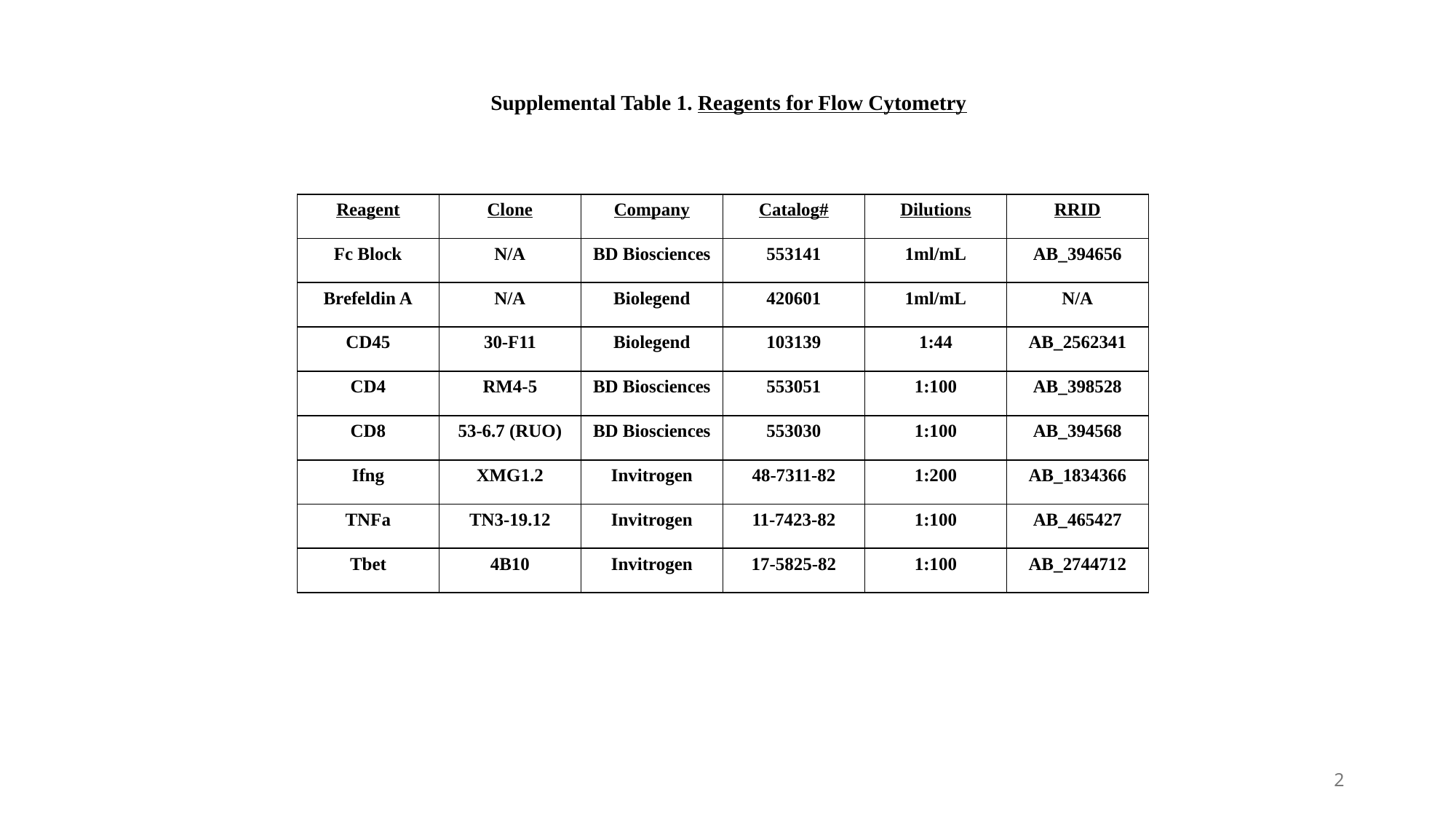

# Supplemental Table 1. Reagents for Flow Cytometry
| Reagent | Clone | Company | Catalog# | Dilutions | RRID |
| --- | --- | --- | --- | --- | --- |
| Fc Block | N/A | BD Biosciences | 553141 | 1ml/mL | AB\_394656 |
| Brefeldin A | N/A | Biolegend | 420601 | 1ml/mL | N/A |
| CD45 | 30-F11 | Biolegend | 103139 | 1:44 | AB\_2562341 |
| CD4 | RM4-5 | BD Biosciences | 553051 | 1:100 | AB\_398528 |
| CD8 | 53-6.7 (RUO) | BD Biosciences | 553030 | 1:100 | AB\_394568 |
| Ifng | XMG1.2 | Invitrogen | 48-7311-82 | 1:200 | AB\_1834366 |
| TNFa | TN3-19.12 | Invitrogen | 11-7423-82 | 1:100 | AB\_465427 |
| Tbet | 4B10 | Invitrogen | 17-5825-82 | 1:100 | AB\_2744712 |
2

## Slide 3
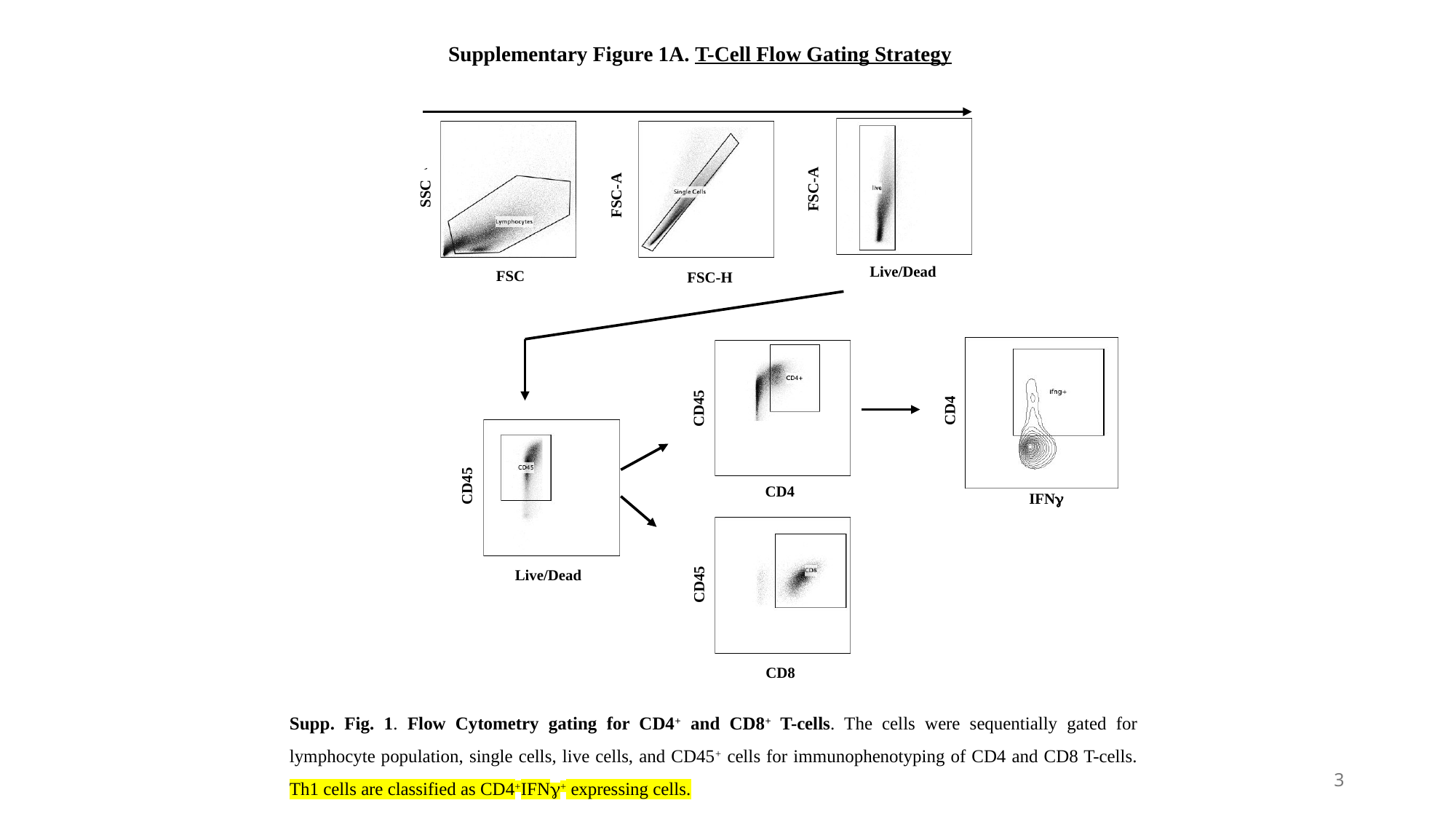

# Supplementary Figure 1A. T-Cell Flow Gating Strategy
FSC-A
SSC
FSC-A
Live/Dead
FSC
FSC-H
CD45
CD4
CD45
CD4
IFNg
Live/Dead
CD45
CD8
Supp. Fig. 1. Flow Cytometry gating for CD4+ and CD8+ T-cells. The cells were sequentially gated for lymphocyte population, single cells, live cells, and CD45+ cells for immunophenotyping of CD4 and CD8 T-cells. Th1 cells are classified as CD4+IFNg+ expressing cells.
3

## Slide 4
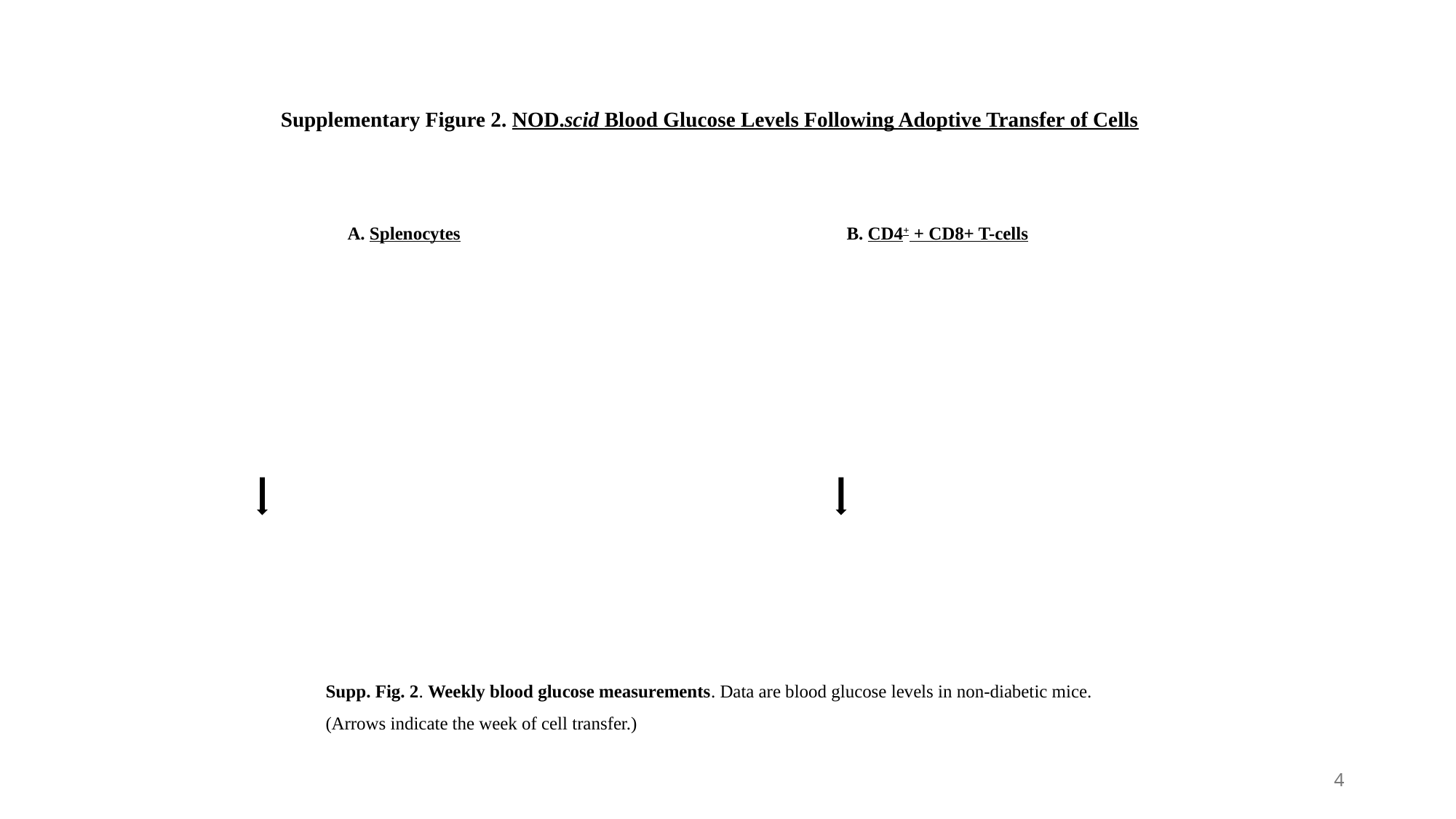

# Supplementary Figure 2. NOD.scid Blood Glucose Levels Following Adoptive Transfer of Cells
A. Splenocytes
B. CD4+ + CD8+ T-cells
Supp. Fig. 2. Weekly blood glucose measurements. Data are blood glucose levels in non-diabetic mice.
(Arrows indicate the week of cell transfer.)
4
